# Effects of Dietary Carbohydrate and Lipid Concentrations on Growth Performance, Feed Utilization, Glucose, and Lipid Metabolism in Two Strains of Gibel Carp

**DOI:** 10.3389/fvets.2019.00165

**Published:** 2019-05-29

**Authors:** Hongyan Li, Wenjie Xu, Junyan Jin, Xiaoming Zhu, Yunxia Yang, Dong Han, Haokun Liu, Shouqi Xie

**Affiliations:** ^1^State Key Laboratory of Freshwater Ecology and Biotechnology, Institute of Hydrobiology, Chinese Academy of Sciences, Wuhan, China; ^2^College of Advanced Agricultural Sciences, University of Chinese Academy of Sciences, Beijing, China

**Keywords:** carbohydrate, lipid, nutrient metabolism, gibel carp, strain

## Abstract

To test the hypothesis that effects of dietary carbohydrate and lipid concentrations on growth performance, feeding utilization, glucose and lipid metabolism in gibel carp A strain may be differ from F strain, these two strain of gibel carp were fed with one of three different isonitrogenous diets: HCLL (45% carbohydrate, 2% lipid), MCML (30% carbohydrate, 8% lipid), or LCHL (15% carbohydrate, 14% lipid). After 8 weeks, the HCLL-fed fish had the highest hepatosomatic index, hepatic crude lipid levels, and triglyceride levels and lipid retention efficiency. Enhanced lipogenesis and lipid uptake potential were observed in fish fed HCLL and MCML diets. Moreover, increases in glucose transport (*glut2, P* = 0.003) and glycolysis (*gk, P* = 0.012; *6pfk, P* = 0.005) in livers of both strains were induced by the high-carbohydrate diet. Genotype-specific effect was identified on plasma lipid content. Plasma triglyceride levels were also greater in the F strain than in the A strain. Furthermore, the F strain had higher levels of fatty acid β-oxidation and glycolysis compared with the A strain. Nutrient retention was affected (*P* < 0.05) by the interaction between genotype and diet, implied dietary carbohydrate played a vital role in lipid accumulation in gibel carp. As dietary lipids increased, the F strain exhibited better feed utilization and a higher PRE than the A strain. However, the A strain had better growth performance. Overall, the F strain had better glucose uptake, glycolysis potential, and lipid utilization ability than the A strain.

## Introduction

Dietary protein is the most expensive ingredient in manufactured fish food, accounting for more than 60% of the total production cost (1). Fishmeal is considered an ideal protein for aquatic feeds (1). However, fishmeal resources are finite, and fishmeal prices have recently increased as wild fishery populations have declined due to overfishing (2). Therefore, dietary lipids and carbohydrates are of increasing importance to aquaculture, as these nutrients are used as non-protein energy sources in fish feeds and therefore reduce protein requirements (3–5).

Carbohydrate is the least expensive and most readily-available source of dietary energy for aquatic feeds (3, 6). Feeds with proper levels of carbohydrate are more efficiently pelletized, reduce the catabolism of proteins and lipids for energy, and provide metabolites for biological syntheses (7, 8). However, fish feeds with an overabundance of dietary carbohydrate may lead to unwanted fat depositions, suppressed immune function, and reduced health (9–11). Dietary lipids are important for fish growth and development as they provide energy, supply essential fatty acids, and act as vectors for fat-soluble vitamins (12, 13). However, suboptimal and/or excess levels of dietary lipids may have adverse effects on fish growth and immunity (10, 14, 15). Importantly, dietary lipid and carbohydrate could affect utilization of each other in fish as lipid could be converted to glucose via gluconeogenesis and glucose could be deposited as lipids in tissues (16). Therefore, determination of the optimal relative levels of carbohydrates and lipids in fish feeds is of great significance to aquaculture.

Fish growth performance and metabolic efficiency vary depending on the dietary carbohydrate and lipid levels (17–19). Any imbalance in the supply of non-protein energy sources may negatively affect fish growth, conversion efficiency, nutrient retention, and body composition (20). Many previous studies have separately investigated the effects of dietary carbohydrate and lipids on fish physiology (15, 21–23). Until now, the interactions between carbohydrate and lipid levels have been investigated in a few fish species, including red drum (*Sciaenops ocellatus*) (24), rainbow trout (*Oncorhynchus mykiss*) (17) and blunt snout bream (*Megalobrama amblycephala*) (18). However, most studies primarily focused on determining the optimal carbohydrate-to-lipid ratio for growth (9, 16, 19). The effects of different dietary carbohydrate and lipid levels on the molecular glucose and lipid metabolisms in fish have rarely been investigated.

The efficiency of carbohydrate and lipid utilization differs among fish species and genotypes, as does the regulation of the glucose and lipid metabolisms. Previous studies have shown that carbohydrate utilization varies in different strains of salmonids (*Oncorhynchus mykiss*) (25, 26). For example, lipids were metabolized differently in two lines of rainbow trout (*Oncorhynchus mykiss*, Walbaum 1792) divergently selected for muscle fat content: one line (“F”) more effectively stored excess glucose and more efficiently bioconverted fatty acids in the intestine than did the other line (“L”) (27, 28). Different lipid sources also induced genotype-specific effects in two lines of Atlantic salmon (*Salmo salar*): the transcriptional expression levels of genes associated with the lipid metabolism (*ppar*α, *ppar*β, and *srebp-1*) decreased in the “lean” strain fed vegetable oil (29). Thus, the molecular metabolic response to variations in the dietary carbohydrate and lipid levels might differ among fish strains.

Gibel carp (*Carassius gibelio*), an omnivorous freshwater fish, is widely cultivated in China due to its high economic value (30, 31). Gibel carp reproduce both sexually and asexually (32). Gibel carp eggs are activated by heterospecific sperm (gynogenesis), producing all-female strains of offspring (32, 33). The CAS III (A) strain, which grows rapidly, is the most commonly cultured gibel carp strain in China, accounting for ~70% of all gibel carp produced (31, 34). Strain CAS V (F), a relatively new strain, has higher disease resistance and faster growth than the A strain (34). Previous studies have shown that insulin treatment differently affected the glucose and lipid metabolisms in two strains (A strain and DT strain) of gibel carp (31). And the DT cleared excess blood glucose more quickly than did the A strain in a glucose tolerance test (35). In addition, gibel carp displayed different priorities and sensibilities in the mobilization of energy reserves (36). Thus, we put forward the hypothesis that the effects of dietary carbohydrate and lipid concentrations on growth performance, feed utilization, glucose, and lipid metabolism could be different between A strain and F strain. Therefore, in this study, A strain and F strain were fed one of three different isonitrogenous diets: HCLL (High Carbohydrate Low Lipid, 45% carbohydrate, 2% lipid), MCML (Medium Carbohydrate Medium Lipid, 30% carbohydrate, 8% lipid), or LCHL (Low Carbohydrate High Lipid, 15% carbohydrate, 14% lipid). Growth performance, feed utilization, gene expression and enzyme activity involved in glucose and lipid metabolism were compared between these two strains among the three diets. Then, any genotype × diet interactions were assessed.

## Materials and Methods

### Experimental Diets

Three isonitrogenous (32% crude protein) experimental diets were formulated, each containing different proportions of carbohydrates and lipids: HCLL (45% carbohydrate and 2% lipid), MCML (30% carbohydrate and 8% lipid), and LCHL (15% carbohydrate and 14% lipid). The dietary protein was provided by white fishmeal and casein. Cornstarch was used as the carbohydrate source; fish oil and soybean oil (in equal amounts) were used as the lipid source (Table 1). All of the ingredients were well ground, fully mixed, and then pelleted using a granulator (SLP-45, Fishery Mechanical Facility Research Institute, Shanghai, China). Pellets were dried in an oven at 50°C and stored at 4°C.Fatty acid compositions of the experimental diets were presented in Table 2.

**Table 1:** Formulation and chemical composition of experimental diets.

**Ingredients (% dry matter)**	**Diets**		
	**HCLL**	**MCML**	**LCHL**
White fishmeal^a^	15	15	15
Casein^b^	24	24	24
Fish oil^c^: soybean oil (1:1)	0	6	12
Corn starch^d^	45	30	15
Vitamin premix^e^	0.39	0.39	0.39
Choline chloride	0.11	0.11	0.11
Mineral premix^f^	5	5	5
Carboxymethylcellulose sodium	3	3	3
Cellulose	7.5	16.5	25.5
**CHEMICAL COMPOSITION (%)**			
Crude protein	32.50	33.20	32.96
Crude lipid	2.35	8.17	14.47
Starch	38.59	24.91	13.88
Ash	6.10	6.32	6.78
Gross energy (kJ/g)	18.14	19.50	20.86

*HCLL, High Carbohydrate Low Lipid, 45% carbohydrate, 2% lipid; MCML, Medium Carbohydrate Medium Lipid, 30% carbohydrate, 8% lipid; LCHL, Low Carbohydrate High Lipid, 15% carbohydrate, 14% lipid*.

a White Fishmeal, American Seafood Company, Seattle, Washington, USA.

b Casein, Lanzhou Longruan Casein Co. Ltd., Lanzhou, Gansu, China

c Fish oil, Anchovy oil from Peru, purchased from Coland Feed Co., Ltd., Wuhan, China.

e Corn starch, Wuhan Coland Feed Co., Ltd., Wuhan, China.

e Vitamin premix (mg/kg diet), thiamin, 20; riboflavin, 20; pyridoxine, 20; cyanocobalamine, 0.020; folic acid, 5; calcium pantothenate, 50; inositol, 100; niacin, 100; biotin, 0.1; starch, 645.2; ascorbic acid, 100; vitamin A, 110; vitamin D, 20; vitamin E, 50; vitamin K, 10.

f *Mineral salt premix (mg/kg diet): NaCl, 500; MgSO_4_·7H_2_O, 4575.0; NaH_2_PO_4_·2H_2_O, 12500.0; KH_2_PO_4_, 16000.0; Ca(H_2_PO_4_)_2_·H_2_O, 6850.0; FeSO_4_, 1250.0; C_6_H_10_CaO_6_·5H_2_O, 1750.0; ZnSO_4_·7H_2_O, 111.0; MnSO_4_·4H_2_O, 61.4; CuSO_4_·5H_2_O, 15.5; CoSO_4_·6H_2_O, 19.02; KI, 178.33; starch, 6253.33*.

**Table 2:** Fatty acid composition (% of total fatty acids) of the experimental diets.

**Fatty acid (%)**	**Diets**		
	**HCLL**	**MCML**	**LCHL**
C14:0	3.27	4.71	6.48
C15:0	0.36	0.46	0.20
C16:0	13.44	11.05	14.24
C17:0	0.28	0.35	0.39
C18:0	6.40	5.85	4.52
ΣSFA^a^	23.75	22.43	25.83
C16:1n-9	5.71	4.48	6.97
C18:1n-9	36.27	26.21	19.44
C20:1n-9	3.54	2.63	1.77
C22:1n-9	2.71	4.04	1.82
ΣMUFA^b^	48.23	37.35	30.00
C18:2n-6	15.18	24.00	21.31
C18:3n-6	0.23	0.14	0.14
C20:2n-6	0.43	0.35	0.41
C20:4n-6	0.60	0.77	0.49
Σn-6 PUFA^c^	16.43	25.26	22.36
C18:3n-3	0.76	3.37	2.51
C20:5n-3	2.69	4.31	3.56
C22:6n-3	4.72	5.76	6.35
Σn-3 PUFA	8.17	13.43	12.42
n-3/n-6 PUFA	0.50	0.53	0.56

HCLL, High Carbohydrate Low Lipid, 45% carbohydrate, 2% lipid; MCML, Medium Carbohydrate Medium Lipid, 30% carbohydrate, 8% lipid; LCHL, Low Carbohydrate High Lipid, 15% carbohydrate, 14% lipid.

a SFA, saturated fatty acids.

b MUFA, monounsaturated fatty acids.

c *PUFA, polyunsaturated fatty acids*.

### Fish and Feeding Trial

Two strains of juvenile gibel carp (*Carassius gibelio*) were obtained from the Institute of Hydrobiology, Chinese Academy of Sciences (Wuhan, Hubei, China): A strain (initial body weight: 3.01 ± 0.02 g) and F strain (initial body weight: 6.20 ± 0.00 g). All fish were acclimated in a fiberglass tank in an indoor rearing system for 2 weeks prior to experimentation and were fed a balanced mix of the experimental diets three times per day. At the beginning of the experiment, all fish were fasted for 24 h. Then fish were randomly allocated among the three dietary groups, with triplicate tanks per group and 30 juveniles per tank. Fish were fed to apparent satiation three times daily (at 08:30, 13:30, and 18:30). The daily feed supplied was recorded, and uneaten feed was collected 20 min after feeding, and dried (37). Throughout the 8-week experiment, water temperature was 25–27°C, pH was 6.5–7.0, dissolved oxygen was >5 mg L^−1^, ammonia nitrogen concentration was < 0.5 mg L^−1^, and light intensity was 2.21–2.95 μmols^−1^ m^−2^, with a photoperiod of 12 L:12 D. The experimental protocol was approved by the ethics committee of the Institute of Hydrobiology, Chinese Academy of Sciences.

### Sample Collection

At 8 h after the final feeding, fish were anesthetized in diluted MS-222 (0.06 g L^−1^; Sigma, St. Louis, MO, USA), counted, batched-weighed, and sampled. Three fish of both strains at the beginning of the experiment and four fish per tank at the end of the experiment were collected and stored at −20°C for body composition analysis. Three fish per tank were randomly selected for the measurement of body weight and body length. The viscera and hepatopancreas were removed from these fish and weighted to calculate conditional factor (CF), viscerosomatic index (VSI), and hepatosomatic index (HSI). Blood samples were collected from an additional two randomly selected fish per tank, using syringes rinsed with heparin sodium (0.2%). After centrifugation at 3,000 g for 10 min, each plasma sample was immediately frozen and held at −80°C until analysis. The liver and dorsal muscle from these fish were immediately removed on ice, frozen in liquid nitrogen, and stored at −80°C for posterior analysis.

### Chemical Analysis

Proximate compositions of the feeds and fish samples were analyzed following AOAC methods (38). Moisture content was determined by oven (Heating and Drying Oven, XMTD-8222, Jinghong, Shanghai, China) drying at 105°C to constant weight (Method 934.01, AOAO 2003). Ash content was determined via combustion in a muffle furnace (Muffle furnace, Yingshan, Hubei, China) at 550°C to a constant weight [Method 942.05, (38)]. Crude protein content (N × 6.25) was determined after acid digestion with the Kjeldahl method [Method 990.03, (38)], using a 4,800 Kjeltec Analyzer Unit (FOSS Tecator, Haganas, Sweden). Crude lipid content was determined using ether extraction in a Soxtec system (Soxtec System HT6, Tecator, Haganas, Sweden), with diethyl ether as the extraction liquid [Method 920.39, (38)]. The starch content of the diet was measured using the commercial starch content kit (A148-1-1, Jiancheng Bioengineering Institute, Nanjing, China). The liver and muscle were freeze-dried in a freeze dryer (Christ ALPHA 1-4 LD plus, Germany), and the crude lipid contents of the freeze-dried organs were determined using ether extraction in a Soxtec system (Soxtec System HT6, Tecator, Haganas, Sweden), with diethyl ether as the extraction liquid. For the measurement of fatty acid compositions, lipids of the experimental diets were extracted with a chloroform:methanol (2:1 v/v) mixture as described by Folch et al. (39). Fatty acid methyl esters were obtained by transmethylation as described by Christie (40). The fatty acid composition of diets was determined using the gas chromatography (7890A-5975C, Agilents Technologies Inc., Santa Clara, CA, USA). An HP-88 capillary column (60 m × 250 μm × 0.2μm, Agilent 112-8867) was used to provide the fatty acid separation. The column was set at a constant flow rate of 1 mL/min using helium as carrier gas, and the septum purge flow was 3 mL/min. The injection volume was 1.0 μL in splitless mode with the split ratio at 10:1. The temperature of injector, ion source, and transfer line was set at 280 °C, 230 °C and 230 °C, respectively. The total runtime was 46 min and the oven temperature was programed as follows: the initial temperature was 100 °C for 5 min, then increased to 230°C (for 15 min) at 5°C/min. Fatty acids in the diet samples were calculated by the standard curves and fatty acid composition was expressed as percentage of total fatty acids.

Plasma glucose, non-esterified fatty acids (NEFAs), triglycerides, and total cholesterol content were determined using commercial kits (Fujifilm, Wako Pure Chemical Corporation, Osaka, Japan), following the manufacturer's instructions. Plasma low-density lipoprotein cholesterol (LDL-C) and high-density lipoprotein cholesterol (HDL-C) were measured using commercial kits (A113-1 and A112-1; Jiancheng Bioengineering Institute, Nanjing, China). The activity levels of several hepatic enzymes [i.e., hexokinase (HK), pyruvate kinase (PK), phosphoenolpyruvate carboxykinase (PEPCK), lipoprotein lipase (LPL) and hepatic lipase (HL)] were determined using commercially kits (Jiancheng Bioengineering Institute, Nanjing, China). The enzyme levels of acetyl-CoA carboxylase (ACC) and fatty acid synthase (FAS) were determined using commercially available ELISA kit (Jiancheng Bioengineering Institute, Nanjing, China).

### Gene Expression Analysis

Total RNAs were extracted from the hepatopancreas and muscle tissue using TRIzol reagent (Invitrogen, Carlsbad, California, USA), and cDNA was reverse transcribed using an M-MLV First-Strand Synthesis Kit (Invitrogen, Shanghai, China), following the manufacturer's instructions. Quantitative reverse transcription-polymerase chain reaction (RT-PCR) was performed on a LightCycler 480 II (Roche Diagnostics, Basel, Switzerland), using SYBR Green I Master Mix (Roche Diagnostics, Indianapolis, IN, USA). The expression levels of genes associated with the glucose or lipid metabolisms were determined using quantitative real-time RT-PCR. Specific RT-PCR primers for *ppar*α (Peroxisome proliferator-activated receptor alpha; F: GTTCTCAGAAGTGTTTGCGTCC; R: GCACTCCATAGTGGAAACCTGA; Genbank accession number: MK160995) and *atgl* (Adipose triglyceride lipase; F: GTGTACTGTGGCCTGATACCT; R: GCGCAGCTCATGGATGTTGGT; Genbank accession number: MK071344) were designed for this study; remaining primers were from Li et al. (36). The mRNA expression levels in the hepatopancreas and the muscle tissues were calculated as fold-change in expression relative to the housekeeping gene, β*-actin*. Gene expression levels were analyzed in six fish (n = 6). Results were calculated based on the Pfaffl mathematical model (41).

### Calculations and Statistical Analysis

The following variables were calculated:

IBW: Initial body weight;

FBW: Final body weight;

Feed intake (FI, g/fish) = dry feed intake / number of fish;

Feed rate (FR, %BW/d) = 100 × dry feed intake / [days × (IBW + FBW) / 2];

Feed efficiency (FE, %) = (100 × fresh body weight gain) /dry feed intake;

Specific growth rate (SGR, % /d) = 100 × [ln (final weight) - ln (initial weight)] /days;

Condition factor (CF, g/cm^3^) = 100 × (body weight) / (body length)^3^;

Hepatosomatic index (HSI, %) = 100 × liver weight / whole body weight;

Viscerosomatic index (VSI, %) = 100 × viscera weight / whole body weight;

Protein retention efficiency (PRE, %) = (100 × body retained protein) / protein intake;

Lipid retention efficiency (LRE, %) = (100 × body retained lipid) / lipid intake.

All of the data were expressed as the mean of all replicates (n = 6) ± the standard error of the mean (s.e.m.). All of the analyses were performed in SPSS (version 22.0; SPSS Inc., Chicago, IL, USA). Taking the different initial body weights of the experimental fish into consideration, a two-way analysis of covariance (ANCOVA) was performed, with initial body weight as a concomitant variable. All of the data were checked for normality and homogeneity of variances using the Shapiro-Wilk and Leven tests. The linearity of the relationship between the variable and the covariate, and the homogeneity of each regression slope, was determined before running the ANCOVA. Where appropriate, differences between strains or diets were analyzed using one-way ANCOVAs.

## Results

### Growth Performance, Feed Utilization, and Fish Morphological Indices

As shown in Table 3, feed intake was 24.6% greater (*P* < 0.001) in F strain compared to A strain. We observed interactions between genotype and diet with respect to FR (*P* < 0.001) and FE (*P* < 0.001) (Table 3). The A strain had a higher (*P* < 0.001) FR than the F strain, regardless of the dietary carbohydrate and lipid concentration. In contrast, the FE of the F strain increased with the decreasing concentration of carbohydrate and increasing concentration of lipid. The SGR of the A strain was higher (*P* < 0.001) than that of the F strain, regardless of diet. No interactions were identified in CF, HSI, or VSI, but the HSI of the F strain was greater (*P* = 0.032) than that of the A strain irrespective of diet (Table 3). In addition, fish fed the HCLL and MCML diets had higher (*P* = 0.022) HSIs than did the LCHL-fed fish.

**Table 3:** Effects of dietary carbohydrate and lipid levels on growth performance, feed utilization, and morphological indices in two strains of gibel carp.

**Diet**	**Strain**	**IBW (g)^**1**^**	**FBW (g)^**2**^**	**FI (g/fish)^**3**^**	**FR (%BW/d)^**4**^**	**FE (%)^**5**^**	**SGR (% /d)^**6**^**	**CF (g/cm^**3**^)^**7**^**	**HSI (%)^**8**^**	**VSI (%)^**9**^**
**HCLL**	A	3.02 ± 0.02	18.24 ± 0.42	24.13 ± 0.46	4.06 ± 0.06^e^	63.04 ± 0.90^a^	3.21 ± 0.05	3.37 ± 0.14	5.46 ± 0.18	14.36 ± 0.22
	F	6.19 ± 0.00	25.55 ± 1.44	29.60 ± 1.72	3.33 ± 0.03^c^	65.25 ± 1.27^a^	2.52 ± 0.11	3.20 ± 0.17	6.24 ± 0.16	15.15 ± 0.09
**MCML**	A	3.01 ± 0.01	19.25 ± 0.22	22.89 ± 0.55	3.67 ± 0.13^d^	71.03 ± 1.29^b^	3.31 ± 0.03	3.39 ± 0.18	5.31 ± 0.39	15.08 ± 0.73
	F	6.21 ± 0.01	29.78 ± 1.37	30.29 ± 1.11	3.01 ± 0.07^b^	77.69 ± 1.89^c^	2.79 ± 0.09	3.53 ± 0.08	7.03 ± 0.63	15.73 ± 0.57
**LCHL**	A	3.01 ± 0.02	17.43 ± 1.23	22.50 ± 1.45	3.93 ± 0.11^e^	63.94 ± 2.04^a^	3.13 ± 0.14	3.51 ± 0.06	4.60 ± 0.74	15.70 ± 0.91
	F	6.21 ± 0.01	28.78 ± 0.86	26.73 ± 0.91	2.73 ± 0.06^a^	84.46 ± 0.28^d^	2.74 ± 0.05	3.26 ± 0.18	4.85 ± 0.37	15.55 ± 0.24
**MEANS OF MAIN EFFECT**										
**Diet**										
HCLL									5.85 ± 0.21^Y^	
MCML									6.17 ± 0.51^Y^	
LCHL									4.73 ± 0.37^X^	
**Strain**										
A		3.01 ± 0.01^A^	18.31 ± 0.46^A^	23.17 ± 0.65^A^			3.22 ± 0.05^B^		5.12 ± 0.27^A^	
F		6.20 ± 0.00^B^	28.04 ± 0.89^B^	28.87 ± 0.65^B^			2.69 ± 0.06^A^		6.04 ± 0.27^B^	
***P***	Strain	<0.001	<0.001	<0.001	<0.001	<0.001	<0.001	0.453	0.032	0.352
	Diet	0.966	0.076	0.136	<0.001	<0.001	0.134	0.496	0.022	0.294
	S × D	0.481	0.162	0.395	<0.001	<0.001	0.266	0.387	0.309	0.659

HCLL, High Carbohydrate Low Lipid, 45% carbohydrate, 2% lipid; MCML, Medium Carbohydrate Medium Lipid, 30% carbohydrate, 8% lipid; LCHL, Low Carbohydrate High Lipid, 15% carbohydrate, 14% lipid.

1 IBW, Initial body weight.

2 FBW, Final body weight.

3 Feed intake.

4 FR, Feed rate.

5 FE, Feed efficiency.

6 SGR, Specific growth rate.

7 CF, Condition factor.

8 HSI, Hepatosomatic index.

9 VSI, Viscerosomatic index.

*Values are expressed as means ± s.e.m. of three replicates. Significant differences among all groups are indicated by different superscripts on each column (a, b, or c) (P < 0.05). The uppercase letters A and B represent significant differences between strains; and the uppercase letters X, Y, and Z represent significant differences among diets (P < 0.05)*.

### Chemical Composition and Nutrient Retention

No strain × diet interactions were identified with respect to whole-body crude protein, crude lipid, moisture, or ash content (*P* > 0.05; Table 4). However, the dietary carbohydrate and lipid concentration affected crude protein (*P* = 0.004) and ash content (*P* = 0.017): fish fed the HCLL diet had higher crude protein and ash levels than did the fish fed with the MCML or the LCHL diet, regardless of genotype (*P* < 0.05). In addition, dietary carbohydrate and lipid concentration and genotype had interactive effects on PRE (*P* < 0.001) and LRE (*P* = 0.026). The PRE of the A strain was lowest in fish fed the LCHL diet, while the PRE of the F strain was highest in fish fed the LCHL diet. In both strains, LRE declined (*P* < 0.001) with the decreasing concentration of carbohydrate and increasing concentration of lipid. Fish fed the HCLL diet had higher (*P* = 0.030) hepatic lipid levels than fish fed the LCHL group, irrespective of genotype (Table 4). With the decreasing concentration of carbohydrate and increasing concentration of lipid, muscle lipid contents increased in the A strain and decreased in the F strain (Table 4).

**Table 4:** Effects of dietary carbohydrate and lipid levels on body chemical composition, liver and muscle lipid content, and nutrient retention efficiency in two strains of gibel carp.

**Diet**	**Strain**	**Crude lipid content (%)**			**Crude protein (%)**	**Ash (%)**	**Moisture (%)**	**PRE (%)^**1**^**	**LRE (%)^**2**^**
		**Fish body**	**Liver**	**Muscle**					
**HCLF**	A	8.16 ± 0.41	7.11 ± 1.07	1.86 ± 0.15^a^	18.04 ± 0.97	4.02 ± 0.35	67.71 ± 2.16	35.24 ± 2.49^b^	275.66 ± 17.05^d^
	F	8.75 ± 0.53	7.50 ± 1.90	2.76 ± 0.16^cd^	16.57 ± 1.25	3.85 ± 0.08	68.26 ± 2.01	33.9 ± 0.53^b^	367.09 ± 17.16^e^
**MCMF**	A	8.90 ± 0.35	6.26 ± 0.71	2.89 ± 0.14^d^	14.88 ± 0.19	3.33 ± 0.10	70.8 ± 0.51	35.82 ± 2.45^b^	95.28 ± 4.41^bc^
	F	9.41 ± 0.40	6.02 ± 0.80	2.27 ± 0.24^abc^	14.64 ± 0.13	3.57 ± 0.03	69.8 ± 0.56	38.77 ± 0.55^b^	127.37 ± 5.40^c^
**LCHF**	A	8.71 ± 0.14	3.87 ± 0.69	2.51 ± 0.16^bcd^	14.89 ± 0.12	3.25 ± 0.04	71.16 ± 0.32	28.14 ± 2.49^a^	41.11 ± 3.88^a^
	F	9.03 ± 0.77	4.35 ± 0.75	2.07 ± 0.11^ab^	15.00 ± 0.40	3.73 ± 0.09	69.46 ± 1.07	45.93 ± 0.77^c^	77.53 ± 3.00^b^
**MEANS OF MAIN EFFECT**									
**Diet**							
HCLF			7.31 ± 0.76^Y^		17.30 ± 0.78^Y^	3.93 ± 0.17^Y^			
MCMF			6.14 ± 0.76^XY^		14.76 ±0.12^X^	3.45 ± 0.07^X^			
LCHF			4.11 ± 0.76 ^X^		14.94 ± 0.19^X^	3.49 ± 0.12^X^			
**Strain**							
A									
F									
***P***	Strain	0.241	0.820	0.124	0.350	0.183	0.521	0.001	<0.001
	Diet	0.363	0.030	0.011	0.004	0.017	0.171	0.288	<0.001
	S × D	0.956	0.940	0.009	0.492	0.154	0.691	<0.001	0.026

HCLL, High Carbohydrate Low Lipid, 45% carbohydrate, 2% lipid; MCML, Medium Carbohydrate Medium Lipid, 30% carbohydrate, 8% lipid; LCHL, Low Carbohydrate High Lipid, 15% carbohydrate, 14% lipid.

1 PRE, Protein retention efficiency.

2 LRE, Lipid retention efficiency.

*Values are expressed as means ± s.e.m. of three replicates. Significant differences among all groups are indicated by different superscripts on each column (a, b, or c) (P < 0.05). The uppercase letters A and B represent significant differences between strains; and the uppercase letters X, Y, and Z represent significant differences among diets (P < 0.05)*.

### Plasma and Liver Chemical Compositions

The interactions between strain and dietary carbohydrate and lipid had no effects on levels of plasma glucose, NEFA, total cholesterol, or LDL-C (Table 5). The levels of NEFA and LDL-C in the F strain were higher (*P* < 0.05) than in the A strain, while fish fed the LCHL diet had lower levels of LDL-C than fish fed the other two diets, irrespective of genotype (*P* < 0.05). The interaction between strain and dietary carbohydrate and lipid were identified (*P* < 0.05) on plasma triglycerides and HDL-C content. The F strain had higher triglyceride (*P* < 0.001) and HDL-C levels (*P* = 0.011) than the A strain across all diets, except for HDL-C levels in fish fed the LCHL diet. In the A strain, triglyceride and HDL-C levels did not vary among different dietary carbohydrate and lipid concentration groups. In contrast, triglyceride and HDL-C levels were lowest in the F strain fish fed the LCHL diet. Hepatic triglycerid (TG) and cholesterol levels were affected (*P* = 0.001) by the dietary carbohydrate and lipid concentrations (Table 5): the highest TG and cholesterol levels were observed in fish fed the HCLL diet, regardless of strain.

**Table 5:** Effects of dietary carbohydrate and lipid levels on plasma and liver metabolite levels in two strains of gibel carp.

**Diet**	**Strain**	**Plasma**							**Liver**	
		**Glucose (mmol/L)**	**Triglycerides (mmol/L)**	**NEFA (mEq/L)^**1**^**	**Cholesterol (mmol/L)**	**LDL-C (mmol/L)^**2**^**	**HDL-C (mmol/L)^3^**		**Triglycerides (mg/g)**	**Cholesterol (mg/g)**
**HCLL**	A	4.05 ± 0.24	3.67 ± 0.19^a^	0.30 ± 0.05	7.14 ± 0.73	7.84 ± 0.41	4.46 ± 0.31^a^		30.73 ± 5.23	5.12 ± 1.02
	F	4.36 ± 0.22	6.89 ± 0.70^c^	0.52 ± 0.08	9.21 ± 0.30	9.70 ± 0.67	6.04 ± 0.25^b^		24.06 ± 3.05	4.32 ± 1.00
**MCML**	A	3.64 ± 0.54	4.24 ± 0.28^ab^	0.30 ± 0.08	7.61 ± 0.78	7.35 ± 0.71	4.24 ± 0.13^a^		11.69 ± 1.07	1.61 ± 0.21
	F	3.78 ± 0.46	6.77 ± 0.55^c^	0.67 ± 0.12	8.05 ± 0.40	9.24 ± 0.80	5.83 ± 0.27^b^		15.81 ± 1.49	2.31 ± 0.31
**LCHL**	A	4.55 ± 0.44	4.86 ± 0.44^ab^	0.33 ± 0.07	7.03 ± 0.42	6.62 ± 0.56	5.03 ± 0.18^ab^		10.25 ± 2.56	1.49 ± 0.38
	F	5.54 ± 0.56	5.3 ± 0.21^b^	0.61 ± 0.08	7.11 ± 0.17	6.87 ± 0.65	4.39 ± 0.77^a^		8.92 ± 2.02	1.22 ± 0.28
**MEANS OF MAIN EFFECT**									
**Diet**									
HCLL						8.77 ± 0.47^Y^			27.40 ± 2.11^Y^	4.72 ± 0.45^Y^
MCML						8.30 ± 0.58^Y^			13.75 ± 2.11^X^	1.96 ± 0.45^X^
LCHL						6.74 ± 0.41^X^			9.58 ± 2.20^X^	1.36 ± 0.47^X^
**Strain**									
A				0.31 ± 0.04^A^		7.27 ± 0.33^A^				
F				0.60 ± 0.05^B^		8.60 ± 0.49^B^				
***P***	Strain	0.764	<0.001	<0.001	0.054	0.016	0.011		0.596	0.818
	Diet	0.385	0.648	0.618	0.121	0.010	0.375		0.001	0.001
	S × D	0.857	0.013	0.670	0.146	0.357	0.008		0.197	0.503

HCLL, High Carbohydrate Low Lipid, 45% carbohydrate, 2% lipid; MCML, Medium Carbohydrate Medium Lipid, 30% carbohydrate, 8% lipid; LCHL, Low Carbohydrate High Lipid, 15% carbohydrate, 14% lipid.

1 NEFA, nonesterified fatty acids.

2 LDL-C, low-density lipoprotein cholesterol.

3 HDL-C, high-density lipoprotein cholesterol.

*Values are expressed as means ± s.e.m. (n = 6). Significant differences among all groups are indicated by different superscripts on each column (a, b, or c) (P < 0.05). The uppercase letters A and B represent significant differences between strains; and the uppercase letters X, Y, and Z represent significant differences among diets (P < 0.05)*.

### Gene Expression Levels

**Figure 1:**
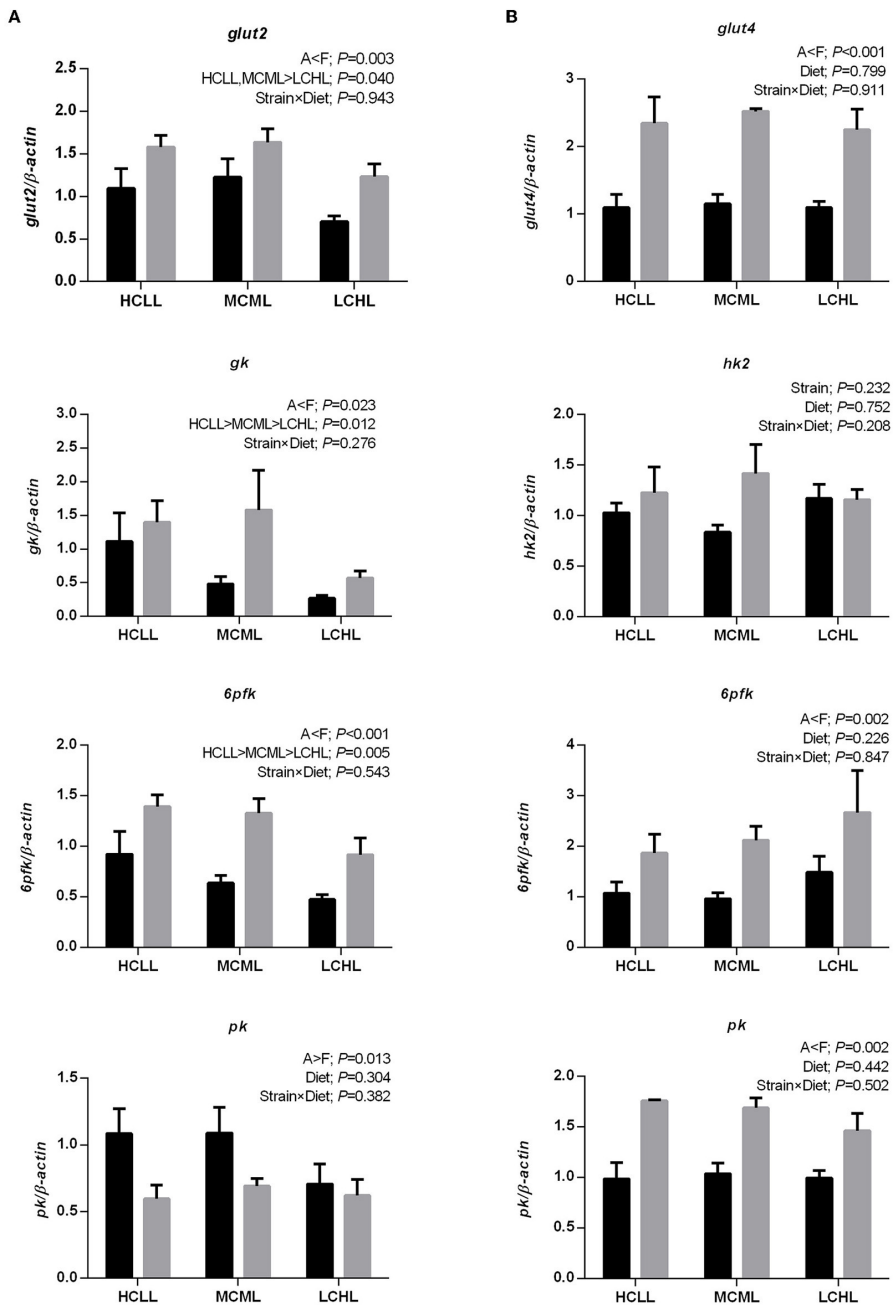
Gene expression levels of selected glucose transporter and glycolytic enzymes in the liver **(A)** and muscle **(B)** of the A strain (black bars) and the F strain (gray bars) fed the HCLL (High Carbohydrate Low Lipid, 45% carbohydrate, 2% lipid), MCML (Medium Carbohydrate Medium Lipid, 30% carbohydrate, 8% lipid), or LCHL (Low Carbohydrate High Lipid, 15% carbohydrate, 14% lipid) diet. Measurements were taken 8 h after the last feeding. Glucose transporter type 2 (*glut2*), glucose transporter type 4 (*glut4*), glucokinase (*gk*), hexokinase 2 (*hk2*), 6-phosphofructokinase (*6pfk*), and pyruvate kinase (*pk*) mRNA levels were measured using real-time quantitative RT-PCR. Results represent the mean ± s.e.m (*n* = 6). Significance was determined using a two-way ANOVA (*P* < 0.05), followed by the Student-Newman-Keuls multiple comparison test.

**Figure 2:**
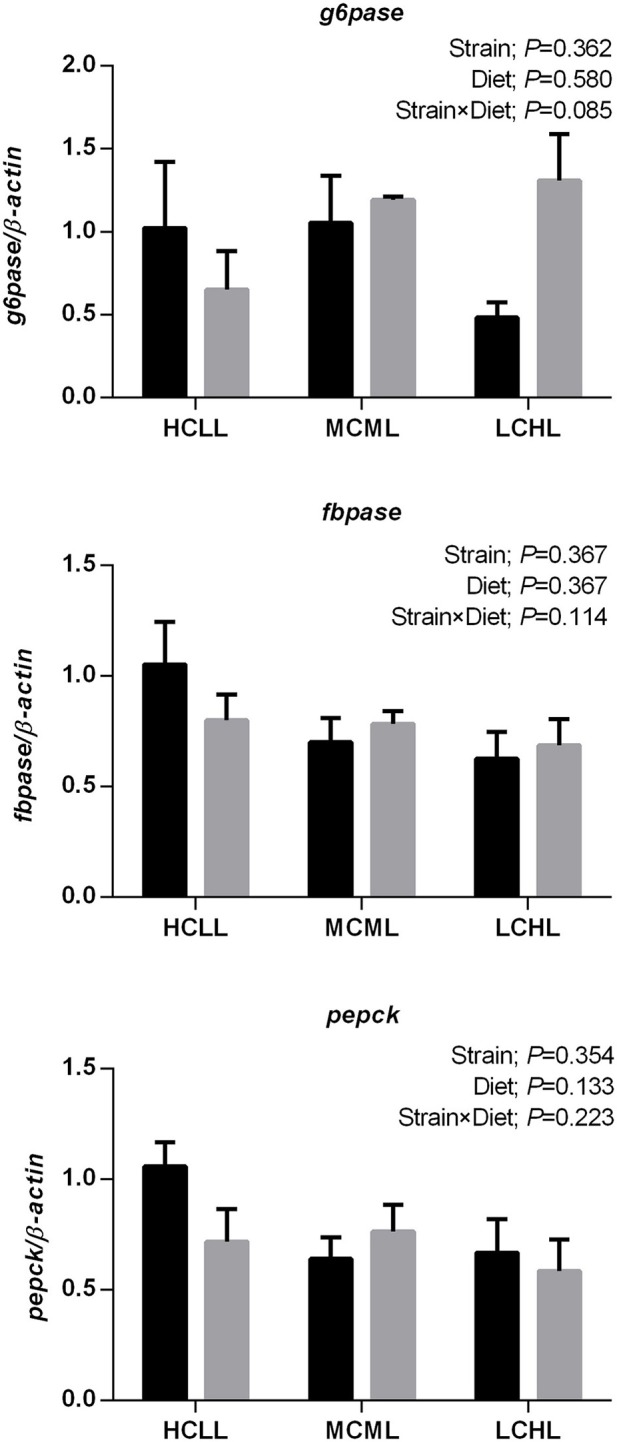
Gene expression levels of selected gluconeogenesis enzymes in the liver of the A strain (black bars) and the F strain (gray bars) fed the HCLL (High Carbohydrate Low Lipid, 45% carbohydrate, 2% lipid), MCML (Medium Carbohydrate Medium Lipid, 30% carbohydrate, 8% lipid), or LCHL (Low Carbohydrate High Lipid, 15% carbohydrate, 14% lipid) diet. Measurements were taken 8 h after the last feeding. Glucose-6-phosphatase (*g6pase*), fructose 1,6-bisphosphatase (*fbpase*), and phosphoenolpyruvate carboxykinase (*pepck*) mRNA levels were measured using real-time quantitative RT-PCR. Results represent the mean ± s.e.m (*n* = 6). Significance was determined using a two-way ANOVA (*P* < 0.05), followed by the Student-Newman-Keuls multiple comparison test.

The transcription levels of *glut2* (*P* = 0.040), *gk* (*P* = 0.012), and *6pfk* (*P* = 0.005) in the liver were lower in fish of both strains fed the low carbohydrate diet as compared to fish fed the other two diets (Figure 1A), but the varying dietary carbohydrate and lipid level had no impact on the expression of genes involved in glucose transport and glycolysis in the muscle of either strain (Figure 1B). The transcription levels of genes associated with glycolysis were higher in both the liver (*gk, P* = 0.023; *6pfk, P* = 0.001) and the skeletal muscle (*6pfk, P* = 0.002; *pk, P* = 0.002) of the F strain as compared to the A strain, with the exception of *pk* transcription in the liver and *hk2* transcription in the muscle. The mRNA expression levels of the hepatic gluconeogenesis enzymes *g6pase, fbpase*, and *pepck* were not affected by the varying dietary carbohydrate and lipid concentration in either strain (Figure 2).

**Figure 3:**
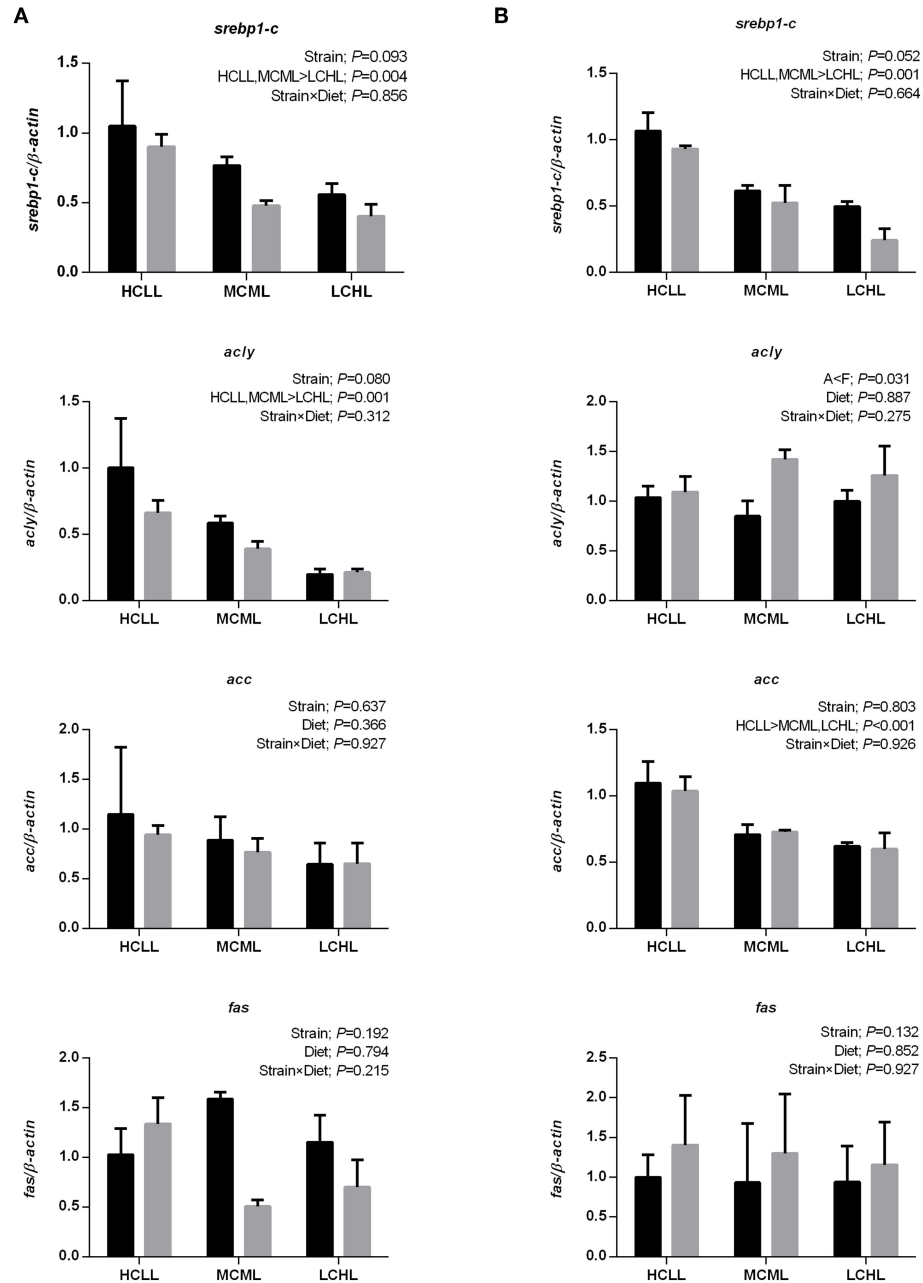
Gene expression levels of selected enzymes and transcription factors associated with nicotinamide adenine dinucleotide phosphate (NADPH) generation and lipogenesis in the liver **(A)** and muscle **(B)** of the A strain (black bars) and the F strain (gray bars) fed the HCLL (High Carbohydrate Low Lipid, 45% carbohydrate, 2% lipid), MCML (Medium Carbohydrate Medium Lipid, 30% carbohydrate, 8% lipid), or LCHL (Low Carbohydrate High Lipid, 15% carbohydrate, 14% lipid) diet. Measurements were taken 8 h after the last feeding. Sterol regulatory element binding protein 1- c (*srebp1-c*), ATP citrate lyase (*acly*), acetyl-CoA carboxylase (*acc*), and fatty acid synthase (*fas*) mRNA levels were measured using real-time quantitative RT-PCR. Results represent the mean ± s.e.m (*n* = 6). Significance was determined using a two-way ANOVA (*P* < 0.05), followed by the Student-Newman-Keuls multiple comparison test.

In both strains, transcriptional factor *srebp1-c* was upregulated in the liver (*P* = 0.004, Figure 3A) and the skeletal muscle (*P* = 0.001, Figure 3B) with the increasing concentration of dietary carbohydrate and decreasing concentration of lipid. Similarly, mRNA expression of *acly* in the liver (*P* = 0.001) and *acc* in the muscle (*P* < 0.001) of both strains increased with the increasing concentration of dietary carbohydrate and decreasing concentration of lipid. The expression of *acly* in the muscle of the F strain was higher (*P* = 0.031) than in the A strain, irrespective of diet. However, the transcriptional expression of *acc* in the liver and *fas* in both tissues were not affected by variations among treatments.

**Figure 4:**
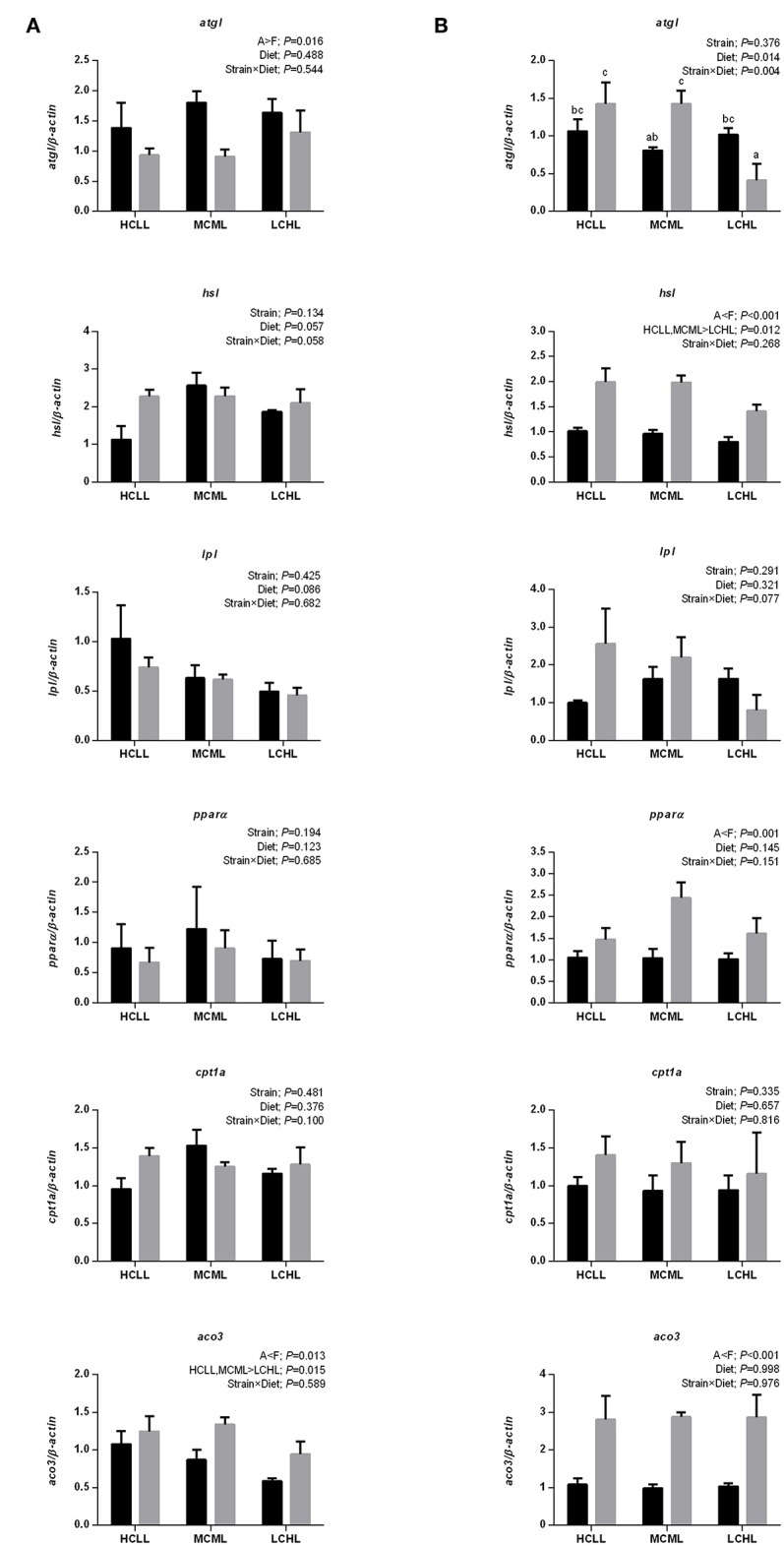
Gene expression of selected enzymes associated with lipolysis and fatty acid oxidation in the liver **(A)** and muscle **(B)** of A strain (black bars) and F strain (gray bars) fed the HCLL (High Carbohydrate Low Lipid, 45% carbohydrate, 2% lipid), MCML (Medium Carbohydrate Medium Lipid, 30% carbohydrate, 8% lipid), or LCHL (Low Carbohydrate High Lipid, 15% carbohydrate, 14% lipid) diet. Measurements were taken 8 h after the last feeding. Lipolysis, including the expression of adipose triglyceride lipase (*atgl*), hormone-sensitive lipase (*hsl*), and lipoprotein lipase (*lpl*) were measured using real-time quantitative RT-PCR. And fatty acid oxidation, as indicated by the expression of peroxisome proliferator-activated receptor alpha (*ppar*α), carnitine palmitoyl transferase 1A (*cpt1a*), and acyl-CoA oxidase 3 (*aco3*) were also measured using the same methods. Results represent the mean ± s.e.m (*n* = 6). Significance was determined using a two-way ANOVA (*P* < 0.05), followed by the Student-Newman-Keuls multiple comparison test.

The mRNA expression levels of genes associated with hepatic lipolysis (*hsl* and *lpl*) and fatty acid oxidation (*ppar*α and *cpt1a*) did not vary with diet in either strain (Figure 4A). However, as compared to A strain, *hsl* and *ppar*α were more transcriptionally abundant in the muscle (*hsl, P* < 0.001; *ppar*α, *P* = 0.001) of the F strain, and *aco3* was more transcriptionally abundant in the liver (*P* = 0.013) and the muscle (*P* < 0.001) of the F strain (Figure 4B). The mRNA expression of *atgl* was higher in the muscle of the F strain as compared to the A strain across all diets except for LCHL. In contrast, *atgl* expression in the liver of the A strain was higher (*P* = 0.016) than in the liver of the F strain. Thus, *hsl* was downregulated (*P* = 0.012) in the muscle and *aco3* was downregulated (*P* = 0.015) in the liver of fish fed the LCHL diet, irrespective of genotype.

### Enzyme Activity

Dietary carbohydrate and lipid affected the activity levels of enzymes associated with glycolysis (HK, *P* = 0.040) and lipid uptake (LPL, *P* = 0.001) in the liver irrespective of genotype (Table 6). The lowest HK and LPL activities were observed in the fish fed LCHL diet. Similarly, the lowest concentrations of enzymes involved in *de novo* fatty acid synthesis (ACC and FAS) were also found in the fish fed the LCHL diet. Neither genotype nor the dietary carbohydrate and lipid affected PK, PEPCK and HL activity levels. A strain displayed higher (*P* = 0.039) levels of LPL activity than the F strain, irrespective of diet.

**Table 6:** Effects of dietary carbohydrate and lipid levels on the activity levels or concentrations of enzymes involved in carbohydrate and lipid metabolism in two strains of gibel carp.

**Diet**	**Strain**	**HK (U/gprot)^**1**^**	**P (U/gprot)^**2**^**	**PEPCK (U/mgprot)^**3**^**	**HL (U/mgprot)^**4**^**	**LPL (U/mgprot)^**5**^**	**ACC (ng/mgprot)^**6**^**	**FAS (ng/mgprot)^**7**^**
**HCLL**	A	7.63 ± 2.06	115.6 ± 18.42	5.57 ± 1.33	1.98 ± 0.20	2.15 ± 0.39	0.32 ± 0.08	3.26 ± 0.79
	F	8.63 ± 3.69	99.84 ± 9.83	4.33 ± 0.47	1.59 ± 0.17	1.92 ± 0.32	0.31 ± 0.05	4.09 ± 0.81
**MCML**	A	7.91 ± 6.29	72.05 ± 15.41	3.96 ± 0.27	1.77 ± 0.47	2.20 ± 0.22	0.28 ± 0.04	4.70 ± 1.51
	F	13.6 ± 7.15	102.49 ± 11.46	4.52 ± 0.22	1.97 ± 0.91	1.15 ± 0.09	0.26 ± 0.04	4.05 ± 0.49
**LCHL**	A	6.58 ± 1.97	88.18 ± 25.41	3.79 ± 0.39	0.86 ± 0.14	0.97 ± 0.15	0.09 ± 0.02	1.51 ± 0.30
	F	6.59 ± 2.64	70.26 ± 19.12	3.57 ± 0.80	1.29 ± 0.42	0.86 ± 0.26	0.15 ± 0.02	1.97 ± 0.35
**MEANS OF MAIN EFFECT**								
**Diet**							
HCLL		8.13 ± 1.41^Y^				2.04 ± 0.19^Y^	0.31 ± 0.03^Y^	3.67 ± 0.57^Y^
MCML		10.75 ± 1.50^Y^				1.68 ± 0.18^Y^	0.27 ± 0.03^Y^	4.37 ± 0.57^Y^
LCHL		6.59 ± 1.63^X^				0.91 ± 0.19^X^	0.12 ± 0.03^X^	1.74 ± 0.57^X^
**Strain**							
A						1.77 ± 0.15^B^		
F						1.31 ± 0.15^A^		
***P***	Strain	0.220	0.940	0.550	0.840	0.039	0.808	0.754
	Diet	0.040	0.240	0.130	0.205	0.001	<0.001	0.009
	S × D	0.390	0.260	0.350	0.697	0.154	0.668	0.641

HCLL, High Carbohydrate Low Lipid, 45% carbohydrate, 2% lipid; MCML, Medium Carbohydrate Medium Lipid, 30% carbohydrate, 8% lipid; LCHL, Low Carbohydrate High Lipid, 15% carbohydrate, 14% lipid.

1 HK, hexokinase.

2 PK, pyruvate kinase.

3 PEPCK, phosphoenolpyruvate carboxykinase.

4 HL, hepatic lipase.

5 LPL, lipoprotein lipase.

6 ACC, acetyl-CoA carboxylase.

7 FAS, fatty acid synthase.

*Values are expressed as means ± s.e.m. (n = 6). Significant differences among all groups are indicated by different superscripts on each column (a, b, or c) (P < 0.05). The uppercase letters A and B represent significant differences between strains; and the uppercase letters X, Y, and Z represent significant differences among diets (P < 0.05)*.

## Discussion

### Dietary Effects

High concentrations of carbohydrate are known to increase HSI in fish (21, 42). Here, higher (*P* = 0.022) HSIs were observed in both strains of gibel carp fed higher concentrations of dietary carbohydrate, consistent with results for blunt snout bream (*Megalobrama amblycephala*) (42, 43), European sea bass (*Dicentrarchus labrax*) (44), and rainbow trout (*Oncorhynchus mykiss*) (21). Excess carbohydrates are converted into simple sugars by digestion, then into pyruvate by glycolysis; pyruvate is then either oxidized for energy or channeled into pathways for fatty acids synthesis (lipogenesis) when energy is available (45). It was therefore unsurprising that the highest levels of hepatic crude lipids and triglycerides were observed in the HCLL-fed fish across both strains. This result may also explain the higher HSIs identified in these groups. The higher LRE levels observed in the HCLL- and MCML-fed groups suggested that carbohydrates might have been converted into lipids and then accumulated in both strains of gibel carp. The lower LRE levels observed in fish fed the LCHL diet might be because more dietary lipids were used for energy in these fish, decreasing lipid retention efficiency. Thus, the dietary carbohydrate and lipid concentrations played a vital role in nutrient retention, despite the interactions identified between diet and genotype.

Crude lipid content did not vary among diets in either strain of gibel carp. Lipid accumulation is a complex process involving lipid transport, uptake, synthesis, and catabolism (46). In vertebrates like fish, lipoproteins are the primary carriers of lipids through the circulatory system of vertebrates (46). Lipoproteins deliver endogenous and dietary lipids to peripheral tissues, where LPL hydrolyzes the TGs of TG-rich lipoproteins (chylomicrons and VLDL) (47). Fatty acids are then released and taken up by the tissues for oxidation or storage (14). Although the mRNA expression of *lpl* did not vary in the liver or muscle of either strain, the elevated hepatic LPL activity observed in gibel carp fed with the HCLL and MCML diets implied an increase in liver tissue fatty acid uptake in these fish. In addition, lipogenesis potential was increased in the fish fed the HCLL diet, as indicated by the increased abundance of *srebp1-c* and *acly* transcripts in the liver and the increased abundance of *srebp1-c* and *acc* in the muscle. Concurrent with this, higher ACC and FAS levels were observed in HCLL and MCML, confirmed that enhanced lipogenesis in these two groups. Such increases might lead to the accumulation of crude lipids and triglycerides in the livers of these fish. Moreover, the HCLL and MCML diets also promoted lipolysis (i.e., increased *hsl* transcription) in the muscle and fatty acid oxidation (i.e., increased *aco3* transcription) in the liver. Thus, increased lipolysis, lipid uptake (i.e., LPL activity) and the induction of the lipogenesis pathway, was observed in both strains fed the HCLL and MCML diets. This result partially explained the similarity in body lipid content across the two strains of gibel carp and across the different dietary carbohydrate and lipid.

Higher concentrations of carbohydrates increased glucose transportation rate and glycolysis potential, as indicated by the upregulation of the hepatic genes *glut2, gk*, and *6pfk* in both strains of gibel carp. This was consistent with results in rainbow trout (*Oncorhyncus mykiss*), and gilthead sea bream (*Sparus aurata*), where the expression of *gk* (the first step of glycolysis) was dramatically upregulated by a carbohydrate-rich diet (48). In line with this, HK activity in the liver was induced (*P* = 0.040) in fish fed HCLL and MCML diets. In fish, muscle represents approximately half of the body mass, and thus plays an important role in glucose homeostasis (49). Unlike the liver, glucose transport and glycolysis in the fish muscle were unaffected by the dietary concentrations of carbohydrate and lipid. This indicated that the liver might be the first organ to respond to variations in the dietary carbohydrate and lipid in gibel carp. Unlike in rainbow trout, both mRNA and activity levels of enzymes involved in gluconeogenesis were unaltered in both A strain and F strain fed a high carbohydrate diet (27). Plasma glucose levels were unaffected by ingestion of a high carbohydrate diet, suggesting that both gibel carp strains were able to efficiently regulate glycemia. Overall, the stable plasma glucose levels might be due to the increased lipogenesis potential of the liver and the muscle, as well as the lack of alteration in gluconeogenesis.

### Genotype Effect

Here, we found that genetic background affected lipid metabolism. Plasma triglyceride content was higher in the F strain than in the A strain, irrespective of diet, despite the interactions (*P* = 0.013) identified between genotype and diet. Indeed, NEFA (*P* < 0.001) and LDL-C (*P* = 0.016) levels were higher in the F strain as compared to the A strain. With respect to lipogenesis, *acly* was upregulated (*P* = 0.031) in the muscle of the F strain as compared to the A strain, irrespective of diet. As compared to the A strain, the F strain had higher (*P* = 0.016) levels of plasma LDL-C; increased *hsl* and *ppar*α transcription in the muscle; and increased *aco3* transcription in both liver and muscle. This implied that, in the F strain, transportation of hepatic lipids to peripheral tissues was better, providing energy via the fatty acid β-oxidation pathway after hydrolyzation. As compared to the F strain, the A strain had a higher *atgl* expression in the liver and lower *hsl* expression in the muscle, suggesting that the triglyceride hydrolyzation limits in the liver and muscle were not the same between strains. However, LPL activity was higher in the A strain than in the F strain, suggesting better hydrolization ability of triacylglycerols in chylomicrons and VLDL in this strain (50).

We found that the growth performance of A strain was better than that of F strain, irrespective of diet. The higher (*P* < 0.001) FR observed in A strain as compared to F strain might be the major factor responsible for the superior growth performance of this strain. However, the glucose uptake of F strain was better than that of A strain, as indicated by the higher expression levels of *glut2* (*P* = 0.003) in the liver and *glut4* (*P* < 0.001) in the muscle. Furthermore, the transcriptional levels of *gk* (*P* = 0.023) and *6pfk* (*P* < 0.001) in the liver, and *6pfk* (*P* = 0.002) and *pk* (*P* = 0.002) in the muscle of F strain were higher than in A strain, indicating that F strain had a greater glycolysis potential than A strain.

### Genotype-Diet Interactions

The interactions between genotype and diet in gibel carp were not identified on growth performance, proximate composition, or appearance. However, the interactions between genotype and diet existed on nutrient retention [i.e., PRE (*P* < 0.001) and LRE (*P* = 0.026)]. Higher LRE levels were observed in the F strain, implying greater lipid accumulation in this strain than in the A strain. Interestingly, dietary carbohydrate level was more important for lipid accumulation than dietary lipid concentration. The energy supplied by dietary lipids has been shown to effectively spare protein in many fish species (5). In blunt snout bream (*Megalobrama amblycephala*), the activity levels of growth and digestive enzymes increased when dietary lipid content increased from 40 to 70 g kg^−1^, reducing the proportion of dietary protein catabolized for energy (13). Thus, increasing lipid contents may provide adequate energy for the F strain, reducing protein catabolism and contributing to the higher PRE observed in the F strain fed the LCHL diet. The superior feed utilization and the higher protein retention efficiency of the F strain fed a high-lipid diet suggested that the F strain utilized lipids better than the A strain.

In conclusion, dietary carbohydrate and lipid concentration interacted with genotype to some extent. For example, although a high-carbohydrate diet stimulated glycolysis and lipogenesis in both gibel carp strains, the F strain more efficiently utilized dietary lipids than the A strain. This was indicated by the higher levels of plasma lipids and lipoproteins, increased fatty acid oxidation potential in the muscle, and improved nutrient retention in the F strain carp fed a high-lipid diet. Glucose uptake and glycolysis potential were also greater in the F strain as compared to the A strain. Thus, genetic selection might help to breed new gibel carp strains with improved carbohydrate and lipid utilization abilities.

## Data Availability

All datasets generated for this study are included in the manuscript and/or the supplementary files.

## Ethics Statement

The experimental protocol was approved by the ethics committee of the Institute of Hydrobiology, Chinese Academy of Sciences.

## Author Contributions

HoL, JJ, and SX designed the study. HoL performed all the experiments and wrote the paper. WX, XZ, YY, DH, HaL, and SX gave suggestions about paper writing. JJ revised the paper.

### Conflict of Interest Statement

The authors declare that the research was conducted in the absence of any commercial or financial relationships that could be construed as a potential conflict of interest.
